# Editorial: Beta-Amyloid oligomer specific treatments for Alzheimer's disease

**DOI:** 10.3389/fnins.2023.1034158

**Published:** 2023-01-25

**Authors:** Heinz Hillen

**Affiliations:** Independent Researcher, Haßloch, Germany

**Keywords:** Alzheimer's disease, beta amyloid (Aβ), oligomer, homeostasis, therapy

## From cascade to dysfunction hypothesis—Paradigm shift in amyloid beta (Aβ) pathology

From the first report of the dominating strength of Aβ as a target for AD treatment (Glenner and Wong, 1984) based on the discovery of PSEN mutations in Familiar Alzheimer's Disease (FAD), excess production of this peptide has been observed (St George-Hyslop and Petit, 2005).

Hence, complementary to the benign alpha secretase pathway, a simple and obvious amyloid beta hypothesis—the amyloid beta cascade hypothesis was formulated (Hardy and Higgins, 1992). This hypothesis defined the peptide Aβ as the central target to treat Alzheimer's Disease. Consequently, a series of gamma secretase (Coric et al., 2012; Doody et al., 2013) and beta secretase (BACE) inhibitors (Panza et al., 2018; Egan et al., 2019) were invented and clinically profiled, aiming to suppress Aβ generation and subsequent plaques in order to cure this fatal disease. Despite broad chemical diversities, all clinically tested secretase inhibitors not only failed but shared cognitive side effects in clinical trials.

The discovery of amyloid-targeted immunotherapy in the brain of transgenic mice (Schenk et al., 1999) triggered a first wave of active and passive immunotherapies that did not discriminate between the three basic conformational categories of Aβ isoforms; i.e., monomers, oligomers, and fibrils.

Recently, five clinical antibody candidates, namely, bapineuzumab (Salloway et al., 2014), solanezumab (Doody et al., 2014), crenezumab (Cummings et al., 2018), aducanumab (Yuksel et al., 2022), and gantenerumab (Roche, in preparation), which were raised by diverse protocols failed to demonstrate robust clinical efficacy in pivotal clinical studies.

Overall, in the last two decades, meaningful prevention and treatment of amyloid beta (Aβ) pathology in Alzheimer's Disease (AD) have been impaired and delayed by denying the physiological role of the steadily produced nascent Aβ monomer in synaptic processing.

The cross reaction with excess Aβ monomer not only violates the physiology of the nascent peptide but also the pharmacokinetic feasibility. The minor fraction of misfolded species cannot be expected to be eliminated by immunotherapy in the presence of excess Aβ monomer at reasonable doses. In addition, the cross reaction with plaques may pose additional issues. Plaque dissolution is associated with ARIA side effects and also limits dosing. It may also interfere with the natural defense function of microglia, that package amyloid beta into dense plaques (Huang et al., 2021).

**Figure 1:**
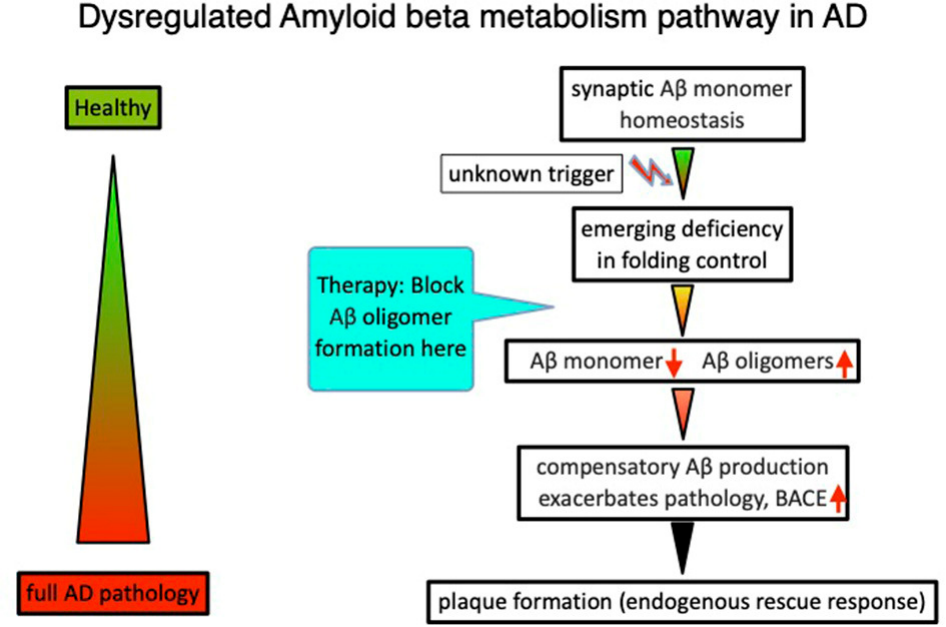
The beta amyloid dysfunction hypothesis (BAD hypothesis) for Alzheimer's disease (Hillen, 2019).

In total, the analysis and interpretation of clinical results from over 20 Aβ targeting compounds, as well as published data on amyloid beta physiology (Copani, 2017), laid the foundations for an alternative beta amyloid dysfunction (BAD) hypothesis, that builds on the essential homeostasis of nascent amyloid beta at synaptic sites. Dysfunction of synaptic performance begins with a deficiency in the folding process, leading to loss of Aβ monomer and the formation of toxic misfolded species (Cline et al., 2018). Deficiencies of nascent Aβ monomer trigger compensatory production *via* BACE elevation in a control loop mode (Figure 1). Thereby, the beta amyloid synthesis enters into a vicious circle because of additional misfolded Aβ production, further overloading the folding control systems (Hillen, 2019). The essential role of the nascent Aβ monomer, and not sAPP or other fragments, was confirmed by investigating human neurons with AD-linked APP-Swedish mutations (Zhou et al., 2022).

## Learning from lecenemab phase 3 data

Recently lecenemab, a soluble protofibril Aβ-targeting antibody (van Dyck et al., 2022) was reported to score an efficacy of ~27% in a pivotal phase 3 AD treatment study. The lecanemab phase 3 data inherently also hide some information about the mode of action. If one calculates the efficacy at all five time points, beginning with 6 months, the efficacy relative to the control group remains constant between 25 and 30% greater across all later time points. Obviously, neither the decreasing amyloid plaque burden nor the changing environment *via* dissolved amyloid beta species during the trial period have an impact on the narrow efficacy range of the antibody. These data are within expectations of the dysfunction hypothesis but are difficult to explain by the amyloid cascade hypothesis, which suggests the deposits as the main role in pathology. According to the cascade hypothesis, the efficacy should depend on the amyloid burden, which is removed from the brain during therapy. This is what we already learned from the AN1792 Active Vaccination trial, where plaques were entirely dissolved during prolonged treatment (Nicoll et al., 2019). By removing amyloid deposits from the brain, the cascade hypothesis would predict an increasing efficacy of antibodies at later time points, administered at constant doses, even if one compensates for age-dependent neuronal loss. However, this is not what has been observed. Maximal activity is in place already at 6 months at the latest, and does not further improve despite lower protofibril levels and/or plaque burden. To further increase the ceiling value above 30% for lecanemab at all stages of this trial, highly isoform-selective, potent, and CNS-penetrable antibodies are needed that precisely target early misfolded conformations. These antibodies should be dosed at a high enough rate to achieve sufficient concentrations at the synapses so as to fully engage Aβ misfolded species produced *in situ*. Drug treatment needs to start at the beginning of pathobiochemistry, before AD is clinically in place, and needs to be chronically maintained. For reasons of cost and convenience, an active vaccination is preferable, since a sufficient antibody concentration can be established at a younger age. A typical concept following this hypothesis is published in WO2016005328, and describes a vaccine based on a mutated, truncated amyloid beta globulomer (Barghorn et al., 2016). This idea of a loss-of-function of the nascent Aβ (Giuffrida et al., 2009) has fundamental consequences for the strategy and design of therapeutic agents such as endogenous or exogenous antibodies and small molecules, targeting early Aβ pathology in AD. The main points to consider are as follows:

1. Focus the binding profile strictly on characteristic epitopes for early misfolded species.
2. Ensure sufficient concentrations of the compound at synaptic sites. Avoid any loss of drug load by unselective binding to any other target, including monomers and plaques.
3. Start prevention or treatment at the earliest time points of pathobiochemistry.
4. No interference with nascent Aβ monomer production and metabolism. Binding to plaques is not the focus of treatment and may lead to unwanted side effects.

This new concept guides us to design potent and highly oligomeric, isoform-selective, and specific therapies, that will enable sufficient CNS drug levels to neutralize early, steadily formed, misfolded species at synaptic sites of MCI or sporadic AD patients' brains. Strict selectivity of antibodies toward the Aβ monomer is essential from a pharmacokinetic perspective to enable effective therapies, since wasting drugs for neutralizing excess monomeric Aβ requires unrealistic high doses, as exemplified by the poor efficacies of monomer and oligomer binding to crenezumab in clinical studies (Cummings et al., 2018).

Some antibodies have been previously described pre-clinically (Hillen et al., 2010; Gibbs et al., 2019).

The contributions in this Research Topic have added preclinical evidence for this concept. The articles by Viola et al. and Krafft et al. describe selective beta amyloid antibodies that may serve as important preclinical and clinical candidates. Two other articles have demonstrated the concept of oligomer-specific treatment not restricted to antibodies. Umeda et al. describe the efficacy of a combination of intranasal Rifampicin and Resveratrol in various transgenic mice strains of neurodegenerative diseases. Wang et al. have shown pre-clinical evidence that TRPV1-mediated microglial activation may be a way to attenuate the pathological oligomeric pathway.

## Future challenges

While these articles may be a good start in a new area of Aβ compounds that satisfy the requirements for high specificity, exclusively directed to early pathological Aβ forms, more efforts are required in further Research Topics to deliver crucial decision criteria for the selection of promising, successful compounds and antibodies. Next to potency, these will provide coverage of early key pathological misfolded Aβ epitopes in the AD brain and pharmacokinetic modeling of CNS available antibody concentrations. Scientists need to agree upon a panel of comparable methods and data to create a safe and convincing translational concept for drug candidates for the clinical treatment of Alzheimer's Disease.

## Author contributions

The author confirms being the sole contributor of this work and has approved it for publication.

## References

[B1] Barghorn S. Hillen H. Striebinger A. Giaisi S. (2016). Abbvie GmbH&CoKG, Immunogenic Products Based on Mutein Amyloid β Amino Acid Sequences and Uses thereof WO2016005328. Wiesbaden: Mainzer Straße.

[B2] Cline E. N. Bicca M. A. Viola K. L. Klein W. L. (2018). The amyloid-β oligomer hypothesis: beginning of the third decade. J. Alzheimers Dis. 64, S567–S610. 10.3233/JAD-179941 29843241 PMC6004937

[B3] Copani A. (2017). The underexplored question of β-amyloid monomers. Eur. J. Pharmacol. 817, 71–75. 10.1016/j.ejphar.2017.05.057 28577967

[B4] Coric V. van Dyck C. H. Salloway S. Andreasen N. Brody M. Richter R. W. (2012). Safety and tolerability of the γ-secretase inhibitor avagacestat in a phase 2 study of mild to moderate Alzheimer's disease. Arch. Neurol. 69, 1430–1440. 10.1001/archneurol.2012.2194 22892585

[B5] Cummings J. L. Cohen S. van Dyck C. H. Brody M. Curtis C. Cho W. (2018), ABBY: A phase 2 randomized trial of crenezumab in mild to moderate Alzheimer's disease. Neurology 90, e1889–e1897. 10.1212/WNL.0000000000005550. 29695589 PMC5962917

[B6] Doody R. S. Aisen P. S. Iwatsubo T. (2013). Semagacestat for treatment of Alzheimer's disease. N. Engl. J. Med. 369, 341–350. 10.1056/NEJMoa1210951 24152267

[B7] Doody R. S. Thomas R. G. Farlow M. Iwatsubo T. Vellas B. Joffe S.. (2014). Phase 3 trials of solanezumab for mild-to-moderate Alzheimer's disease. N. Engl. J. Med. 370, 311–321. 10.1056/NEJMoa1312889 24450890

[B8] Egan M. F. Kost J. Voss T. Mukai Y. Aisen P. S. Cummings J. L. (2019). Randomized trial of verubecestat for prodromal Alzheimer's disease. N. Engl. J. Med. 380, 1408–1420. 10.1056/NEJMoa1812840 30970186 PMC6776078

[B9] Gibbs E. Silverman J. M. Zhao B. Peng X. Wang J. Wellington C. L. (2019). A rationally designed humanized antibody selective for amyloid beta oligomers in Alzheimer'S disease. Sci. Rep. 9, 9870. 10.1038/s41598-019-46306-5 31285517 PMC6614461

[B10] Giuffrida M. L. Caraci F. Pignataro B. Cataldo S. De Bona P. Bruno V. (2009). Beta-amyloid monomers are neuroprotective. J. Neurosci. 29, 10582–10587. 10.1523/JNEUROSCI.1736-09.2009 19710311 PMC6665714

[B11] Glenner G. G. Wong C. W. (1984). Alzheimer's disease: initial report of the purification and characterization of a novel cerebrovascular amyloid protein. Biochem. Biophys. Res. Commun. 120, 885–890. 10.1016/s0006-291x(84)80190-4 6375662

[B12] Hardy J. A. Higgins G. A. (1992). Alzheimer's disease: the amyloid cascade hypothesis. Science 256, 184–185. 10.1126/science.1566067 1566067

[B13] Hillen H. (2019). The beta amyloid dysfunction (BAD) hypothesis for Alzheimer's disease. Front. Neurosci. 13, 1154. 10.3389/fnins.2019.01154 31787864 PMC6853841

[B14] Hillen H. Barghorn S. Striebinger A. Labkovsky B. Müller R. Nimmrich V. (2010). Generation and therapeutic efficacy of highly oligomer-specific beta-amyloid antibodies. J. Neurosci. 30, 10369–10379. 10.1523/JNEUROSCI.5721-09.2010 20685980 PMC6634649

[B15] Huang Y. Happonen K. E. Burrola P. G. O'Connor C. Hah N. Huang L.. (2021). Microglia use TAM receptors to detect and engulf amyloid β plaques. Nat. Immunol. 22, 586–594. 10.1038/s41590-021-00913-5 33859405 PMC8102389

[B16] Nicoll J. A. Buckland G. R. Harrison C. H. Page A. Harris S. Love S.. (2019). Persistent neuropathological effects 14 years following amyloid-β immunization in Alzheimer's disease. Brain 142, 2113–2126. 10.1093/brain/awz142 31157360 PMC6598630

[B17] Panza F. Lozupone M. Solfrizzi V. Sardone R. Piccininni C. Dibello V. (2018). BACE inhibitors in clinical development for the treatment of Alzheimer's disease. Expert Rev. Neurother. 18, 847–857. 10.1080/14737175.2018.1531706 30277096

[B18] Salloway S. Sperling R. Fox N. C. Blennow K. Klunk W. Raskind M.. (2014). Two phase 3 trials of bapineuzumab in mild-to-moderate Alzheimer's disease. N. Engl. J. Med. 370, 322–333. 10.1056/NEJMoa1304839 24450891 PMC4159618

[B19] Schenk D. Barbour R. Dunn W. Gordon G. Grajeda H. Guido T.. (1999). Immunization with amyloid-β attenuates Alzheimer-disease-like pathology in the PDAPP mouse. Nature 400, 173–177. 10.1038/22124 10408445

[B20] St George-Hyslop P. H. Petit A. (2005). Molecular biology and genetics of Alzheimer's disease. C. R. Biol. 328, 119–130. 10.1016/j.crvi.2004.10.013 15770998

[B21] van Dyck C. H. Swanson C. J. Aisen P. Bateman R. J. Chen C. Gee M.. (2022). Lecanemab in early Alzheimer's disease. N. Engl. J. Med. 388, 9–21. 10.1056/NEJMoa2212948 36449413

[B22] Yuksel J. M. Noviasky J. Britton S. (2022). Aducanumab for Alzheimer's disease: summarized data from EMERGE, ENGAGE, and PRIME studies. Sr. Care Pharm. 37, 329–334. 10.4140/TCP.n.2022.329 35879846

[B23] Zhou B. Lu J. G. Siddu A. Wernig M. Südhof T. C. (2022). Synaptogenic effect of APP-Swedish mutation in familial Alzheimer's disease. Sci. Transl. Med. 14, eabn9380. 10.1126/scitranslmed.abn9380 36260691 PMC9894682

